# Regulatory Mechanisms of Hsp90

**DOI:** 10.21767/2471-8084.100030

**Published:** 2017-01-30

**Authors:** Chrisostomos Prodromou

**Affiliations:** Genome Damage and Stability Centre, Science Park Road, Falmer, Brighton, UK

**Keywords:** Chaperones, Co-chaperones, Heat-shock response, HSF1, Hsp90, Post-translational regulation

## Abstract

The ability of Hsp90 to activate a disparate clientele implicates this chaperone in diverse biological processes. To accommodate such varied roles, Hsp90 requires a variety of regulatory mechanisms that are coordinated in order to modulate its activity appropriately. Amongst these, the master-regulator heat shock factor 1 (HSF1) is critically important in upregulating Hsp90 during stress, but is also responsible, through interaction with specific transcription factors (such as STAT1 and Strap/p300) for the integration of a variety of biological signals that ultimately modulate Hsp90 expression. Additionally, transcription factors, such as STAT1, STAT3 (including STAT1-STAT3 oligomers), NF-IL6, and NF-kB, are known to influence Hsp90 expression directly. Co-chaperones offer another mechanism for Hsp90 regulation, and these can modulate the chaperone cycle appropriately for specific clientele. Co-chaperones include those that deliver specific clients to Hsp90, and others that regulate the chaperone cycle for specific Hsp90-client complexes by modulating Hsp90s ATPase activity. Finally, post-translational modification (PTM) of Hsp90 and its co-chaperones helps too further regulate the variety of different Hsp90 complexes found in cells.

## Introduction

**Figure 1:**
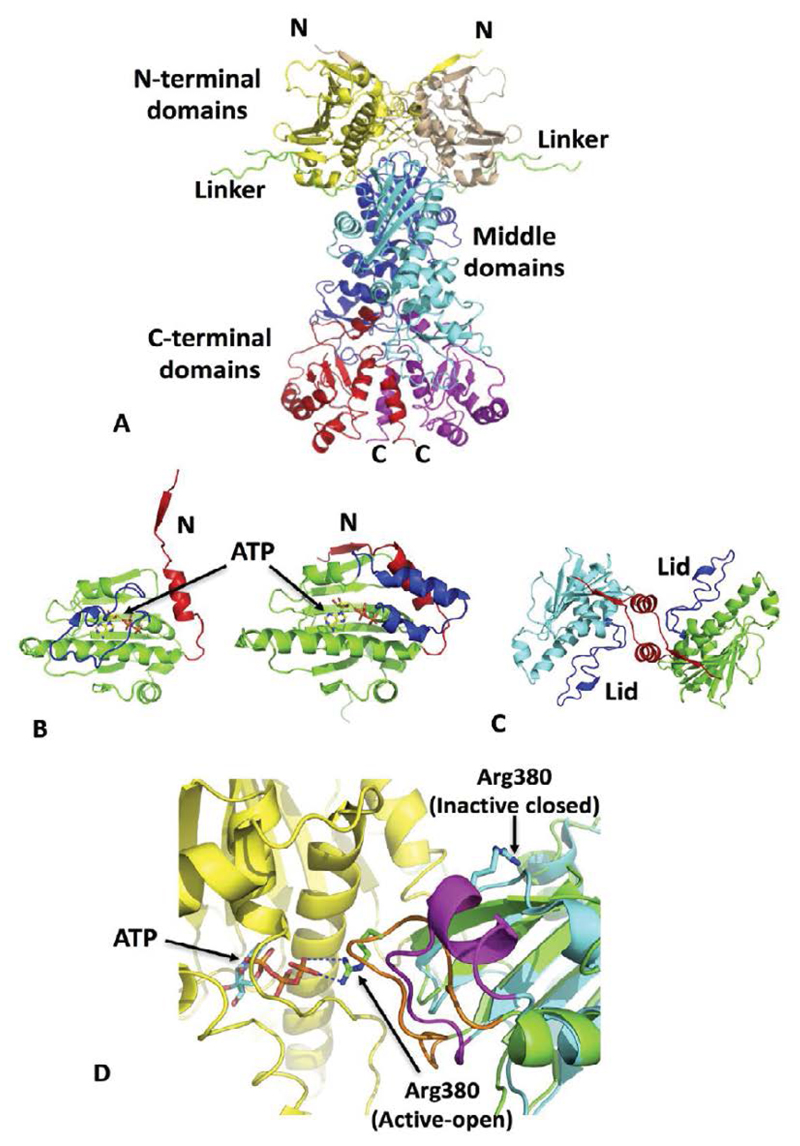
The structure and conformational changes of the Hsp90 chaperone. (A) The closed conformation of the Hsp90 dimer showing transient N-terminal domain (yellow and brown) dimerization. Middle domains are colored blue and cyan while C-terminal domains are shown in red and magenta. The charged linker is shown in green. (B) Conformation of the lid and N-terminal segment of the Hsp90 N-terminal domains. The closed undimerized state is shown in the left-hand panel and the open dimerized state in the right-hand panel. Lids are colored in blue and N-terminal segment in red. (C) The N-terminally dimerized state of Hsp90 (cyan and green). Lids are shown in blue and N-terminal segment in red. N-terminal dimerization involves a series of cooperative structural movements including the N-terminal and lid segments. (D), Catalytic loop conformation of Hsp90. Hsp90 N-terminal domain is shown in yellow. Two superimposed Hsp90 molecules (cyan and green) represent the middle domain. The closed inactive catalytic loop is shown in magenta and the open active state in orange and interacting with bound ATP (stick model). Arg380 of the catalytic loop interacts with ATP only in the active state (or open state). Hydrogen bonds are represented by broken blue lines.

Hsp90α and Hsp90β interact with ˜2000 client proteins [1,2], although only ˜725 of these have been confirmed by direct protein-protein interaction. Hsp90 consists of an N-terminal ATP binding domain, separated from the middle domain by a charged linker (Figure 1). The C-terminal domain is not only responsible for the inherent dimerization of the chaperone, but the conserved MEEVD motif, at its extreme C-terminus, provides the binding site for a variety of TPR domain containing co-chaperones. These include the phosphatase Pp5, immunophilins (for example FKBP51 and FKBP52) and HOP, that aid and modulate its function.

Binding of ATP results in a coordinated restructuring of Hsp90 [3], including the release of an N-terminal domain lid-region, which binds over the N-terminal domain bound ATP and simultaneously provides a platform for N-terminal dimerization (Figure 1). Additionally, the middle-domain catalytic loop of Hsp90 is able to adopt an active conformation by interacting with bound ATP (Figure 1). These structural changes together conspire in a coordinated fashion to form an N-terminally dimerized state that is catalytically active [4–6] (Figure 1). The dimerized state is further stabilized by a β-strand swap involving the N-terminal domains (Figure 1). A detailed description of these changes has been previously described [3,7]. Importantly, the varied structural changes of Hsp90 allow for a variety of regulatory points that can affect the Hsp90 chaperone cycle. These include both PTMs and modulation by co-chaperones. However, the rate-limiting step of the chaperone cycle is determined by the structural rearrangements of Hsp90, which ultimately ends with the hydrolysis of ATP and the disassociation of N-terminal dimerization, and perhaps maturation of a client protein.

## Transcriptional Regulation and Heat Shock Response

HSF1, which is itself a Hsp90 dependent client protein, is the master regulator of the heat shock response (HSR) [8–11] and consequently under strict regulation. Determining the mechanistic action of HSF1 in detail is central to understanding the complex transcriptional regulatory systems of Hsp90. HSF1 is normally associated with Hsp90 in a repressed state, but during stress HSF1 is released [12–14] and homotrimerises [14], whereupon it gains DNA binding activity and is targeted to heat-shock-factor elements (HSE). However, to gain transcriptional activity and transactivation competence [15–23], a series of phosphorylations are required (notably at Ser 230) [24,25], which leads to a rapid up-regulation of Hsp90, Hsp70 and a variety of co-chaperones including Hsp27 and Hsp40 [26]. Additionally, stress is required to overcome a stress-regulated repressive state brought about by specific phosphorylations (Ser 230, Ser 303 and Ser 307) [24,27,28], and HSF1 is known to respond directly to changes in temperature [29–31]. Phosphorylations of HSF1 are also known to be involved in the integration of signals from other signaling pathways [24,25]. For example, phosphorylation at Ser 326 promotes association with Daxx, a mediator of HSF1 activation. Although the phosphorylation of HSF1 is important it appears that the stress-inducible phosphorylation of HSF1 can be uncoupled from its activation [32].

SUMOylation [33–36] and acetylation [37–39] are reported to regulate HSF1. A number of other mechanisms also appear to regulate HSF1, including heat stress, which is directly detected by the regulatory domain of HSF1 [40], by Hsp90 repression of trimeric HSF1 [12,41], and by the inhibition of HSF1 trans-activating activity by Hsp70 and Hsp40. The inhibitory activity of Hsp70 is probably through the recruitment of CoREST (corepressor for element-1-silencing transcription factor), a transcriptional co-repressor of Hsp70 [42,43]. An activator consisting of the eukaryotic elongation factor 1A (eEF1A) together with a constitutively expressed non-coding heat-shock RNA-1 RNA has been reported to upregulate HSF1 [44,45]. The molecular chaperone TriC has also been seen to associate with HSF1 [46], however, the significance of this remains unknown.

**Figure 2:**
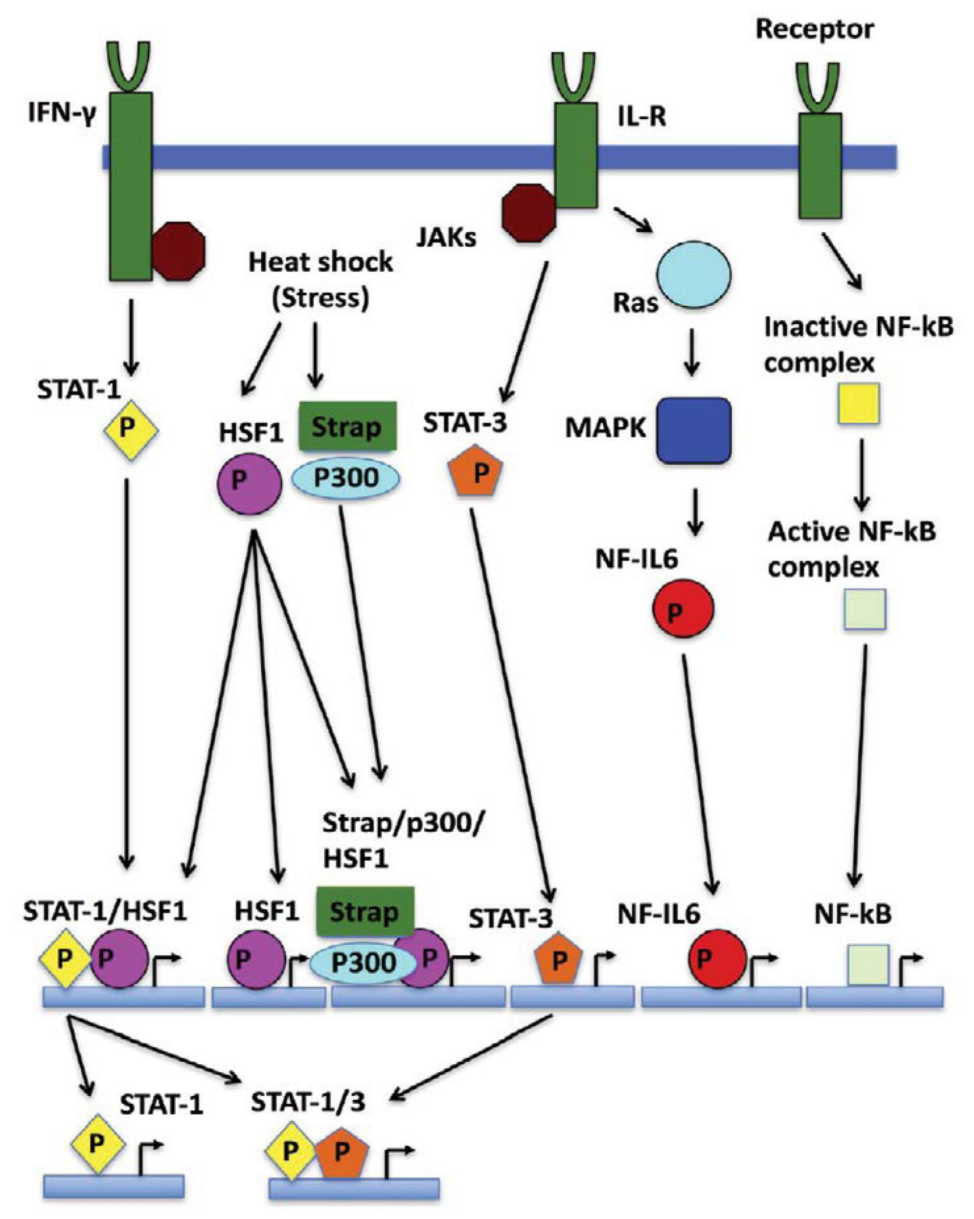
Signal pathway integration that regulates Hsp90 expression. Known signaling pathways that affect expression of Hsp90 and various scenarios for binding. Not shown is the co-activator Daxx, which is known to promote HSF1 activation. [83]. The blue rectangles represent the Hsp90 promotor regions upon which various combinations of transcription factors operate. STAT1 may function independently of HSF1 or with STAT3. IFN-γ, interferon-γ; IL-R, interleukin receptor; JAK, Janus kinase; MAPK, mitogen-activated protein kinase.

Active HSF1 associates with HSE that consist of a number of nGAAn units (reviewed in [47]) where their exact spatial arrangement can influence cooperativity between binding trimers of HSF. The promotor regions of Hsp90 genes are complex providing elements that downregulate and upregulate expression [48] (Figure 2), STAT-1 and STAT-3 binding sites appear to overlap with HSE of HSF1 [47,49,50]. Strong transcriptional activation is seen by the interaction between HSF1 and STAT-1. In contrast, HSF1 and STAT-3 antagonize each other because they appear unable to interact with each other, and thus reduce expression of Hsp90β [49,51]. IL (interleukin)-6 transcription factor NF-IL6 (nuclear factor for IL-6) can also upregulate Hsp90β. On the other hand, Hsp90 β regulation is augmented by the stress-responsive activator of p300, Strap [52], which together form a HSF1 chromatin-associated complex. Since p300 has been reported to possess a histone acetylase activity [53], chromatin acetylation may be a mechanism by which Hsp90 expression is upregulated. The 5'-flanking region of the *HSP90AA1* promoter (but not in *HSP90AB1*) is also bound by NF-κB [54], and the dependence of NF-κB and IKK (inhibitor of NF-κB kinase) on Hsp90 suggests a regulatory loop that can influence a cells response to stress and ultimately its survival.

Clearly, the regulation of transcription of Hsp90 represents a central hub at which diverse signals can be integrated into regulating Hsp90 levels and the HSR (Figure 2). In order to integrate such diverse signals, HSF1 plays a major role and is consequently itself subject to a complex regulatory process, including the ability to to directly sense heat stress.

## Post-Translational Regulation of The Hsp90 Complex

PTM of Hsp90 and its co-chaperones include not only phosphorylation, but also acetylation, methylation, S-nitrosylation, SUMOylation and ubiquitylation and have been reviewed in [55,56]. Such modifications have been shown to be specific to either Hsp90α or Hsp90β [57,58], and can regulate Hsp90 activity either directly or by its interaction with co-chaperones, nucleotides or client protein [57,59–63]. PTM of co-chaperones has been shown to be necessary for the chaperoning of kinase clients [64–66] and Ser 13 dephosphorylation of Cdc37^p50^ by PP5/Ppt1 appears to signal chaperone cycle progression [67]. In contrast, Cdc37^p50^ phosphorylation at Tyr 4 and Tyr 298 appears to disrupt Cdc37^p50^-client association and thus provides directionality to the chaperone cycle [61]. Additionally, Tyr197 phosphorylation of Hsp90 appears to cause Cdc37^p50^ dissociation from Hsp90 [61], whereas Tyr 313 phosphorylation may promote the recruitment of Aha1, both of which stimulate the ATPase activity of Hsp90 and further the chaperoning process. c-Abl kinase has been reported to phosphorylate of Tyr 223 of human Aha1, which appears to differentially affect client protein association [68], however, the same authors reported that Tyr 223 phosphorylation also led to proteasome degradation of Aha1. Tyr 627 phosphorylation of Hsp90α induces client and co-chaperones dissociation, which might signal completion of the kinase chaperone cycle.

The dimerization of Sgt1, another Hsp90 co-chaperone, appears to be influenced by Ser 361 phosphorylation. This in turn affects kinetochore assembly and therefore chromosome segregation in eukaryotic cell division [69]. p23 (cytoplasmic prostaglandin E synthase 3) [70], murine Sti1/HOP and FKBP52 are other Hsp90 co-chaperones that have been shown to be regulated by PTMs, and have roles in a variety of processes including the cell cycle, steroid hormone activation and telomerase maturati [65,71–74].

Clearly, PTM of Hsp90 and its co-chaperones are a major regulatory mechanism of the chaperone cycle, such that the activation of specific client proteins is optimized. This is critically important as the clientele of Hsp90 collectively represent a structurally diverse set of proteins, whose maturation and activation have their own specific requirements.

## Regulation of Hsp90 by Co-Chaperones

The chaperone cycle of Hsp90 is driven by coordinated structural rearrangements following ATP binding, which leads to N-terminal dimerization of Hsp90 [3,5,7,75]. The Co-chaperones HOP, Sgt1 and Cdc37^p50^ are major players in delivering client proteins to the Hsp90 chaperone (see [7,76] for reviews). Cdc37^p50^ and HOP silence Hsp90 ATPase activity and thus facilitate the binding of client protein with Hsp90 [77–79]. In contrast, Sgt1 when in complex with the plant protein Rar1 (CHORD domain containing co-chaperone, also known as Chp1 and melusin in mammals) may form a stable ADP bound complex [80].

Other co-chaperones help to regulate the chaperone cycle by influencing the ATPase activity of Hsp90. For example, Aha1 strongly accelerates the ATPase activity of Hsp90 [6,81] by promoting the rate-limiting structural changes of Hsp90 [3,75]. In contrast, the ATPase activity of Hsp90 is slowed by the co-chaperone Sba1 [81], although the human orthologue p23 shows a more robust inhibition [82]. The slowing of the cycle may be important for generating longer lived client-Hsp90 complexes, which may favor their maturation.

**Figure 3:**
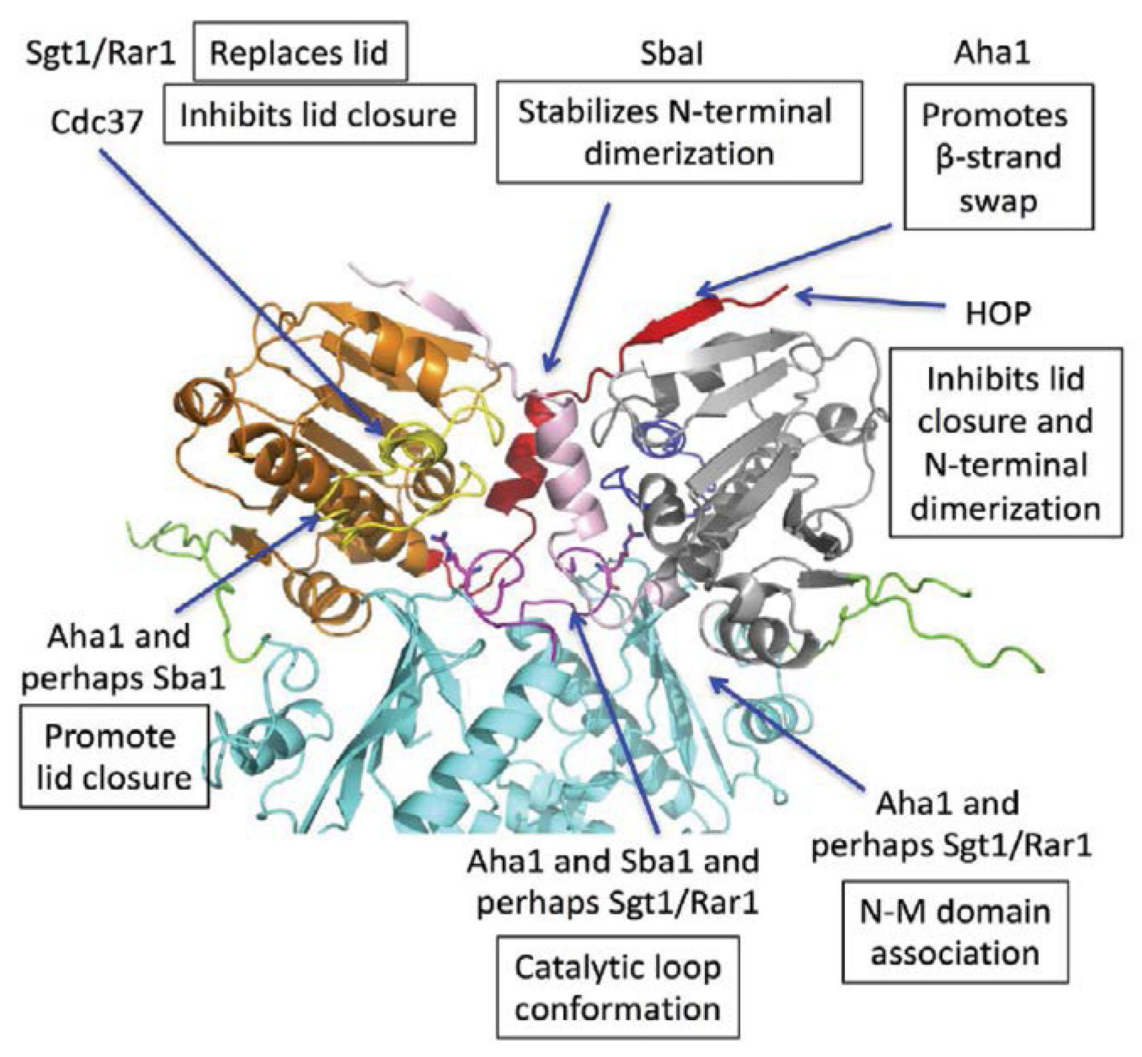
Points of co-chaperone action on structural elements of Hsp90 essential for its ATPase activity. Cdc37^p50^ prevents molecular rearrangement of the lids of Hsp90. HOP may prevent lid closure and N-terminal dimerization probably by interacting with the N-terminal segments of Hsp90. Aha1 appears to interact with all the structural elements leading to a co-operative N-terminally dimerized state of Hsp90. Sba1 can stabilize Hsp90 complexes by reducing the ATPase activity of Hsp90 and it appears to interact with both the lid and N-terminal domains of Hsp90. Sba1 may also modulate the middle domain catalytic loop. Sgt1-Rar1 complex, appear to activate Hsp90 in an open state and convert it to a stable ADP-bound state.

In summary, co-chaperone modulation of the chaperone cycle is clearly an important mechanism by which specific Hsp90 complexes are regulated, and the complex set of structural changes of Hsp90 has allowed a variety of mechanisms to evolve that act upon these critically important points of structural change (Figure 3).

## Conclusion

Many biological processes, including stress adaptation are dependent on Hsp90 and numerous regulatory systems therefore operate to integrate and regulate Hsp90 activity appropriately. As a master regulator of the HSR, HSF1 not only helps to determine Hsp90 levels directly but integrates varied cellular signals into the transcriptional control Hsp90. Direct regulation of the Hsp90 protein involves PTMs, as well as an ability to directly sense heat stress and by direct control through co-chaperone action and its client proteins, which are themselves subject to various regulatory processes. Although some progress has been made in understanding these processes, a substantial amount remains unknown and our knowledge of Hsp90 regulation remains in its infancy. Many of the regulatory enzymes, including phosphatases, kinases, histone deacetylases and histone acetylases remain unknown. Determining how such modifications are translated into coherent regulatory processes will be challenging, but is non-the-less essential to understanding the Hsp90 chaperone cycle and the various biological processes dependent on Hsp90.
